# Two new species of *Otacilia* Thorell, 1897 (Phrurolithidae) from Baima Mountain Nature Reserve, Chongqing, China

**DOI:** 10.3897/BDJ.13.e176953

**Published:** 2025-12-09

**Authors:** Zijian Shi, Luyu Wang, Ziyun Shu, Hua Wang, Yannan Mu

**Affiliations:** 1 Southwest University, Beibei District, Chongqing City, China Southwest University Beibei District, Chongqing City China; 2 Baimashan Forest Farm, Wulong District, Chongqing City, China Baimashan Forest Farm Wulong District, Chongqing City China

**Keywords:** classification, description, taxonomy, morphology

## Abstract

**Background:**

The genus *Otacilia* Thorell, 1897 comprises 141 species worldwide, with 118 (approximately 84%) distributed in China.

**New information:**

Two new species of the genus *Otacilia* are described from Baima Mountain Nature Reserve, Chongqing, China: *Otacilia baimashan* Shi, Wang & Mu, **sp. nov.** (♂♀) and *Otacilia wulong* Shi, Wang & Mu, **sp. nov.** (♂♀). Detailed description, photos of habitus and copulatory organs of the new species are provided.

## Introduction

The Baima Mountain Nature Reserve is situated in eastern Wulong District, Chongqing, and borders Dashahe National Nature Reserve in Guizhou Province. Its vegetation, consisting of evergreen broadleaf and mixed coniferous–broadleaf forests, provides habitat for rare plants (e.g. *Cathaya argyrophylla*, *Davidia involucrata*, *Taxus chinensis*) and animals (e.g. *Chrysolophus pictus*, *Moschus berezovskii*, *Viverricula indica*).

*Otacilia* Thorell, 1897, a genus belonging to the family Phrurolithidae Banks, 1892, contains 141 species around the world, of which 118 species (approximately 84%) are recorded from China (World Sipder Catalog 2025). In China, species of *Otacilia* are predominantly distributed in the southern regions, many being known only from type localities (Mu et al. 2022).

During a survey of Baima Mountain, two undescribed species of *Otacilia* were found and described here: *Otacilia baimashan* Shi, Wang & Mu, sp. nov. (♂♀) and *Otacilia wulong* Shi, Wang & Mu, sp. nov. (♂♀). All specimens of these two new species were collected in leaf litter.

## Materials and methods

All specimens were preserved in 75% ethanol, then examined, photographed and measured using a Leica M205A stereomicroscope equipped with a Leica DFC450 Camera and LAS software (Ver. 4.6). The palps were dissected from the femur. The epigynes were removed and cleared in a pancreatin solution (Álvarez-Padilla and Hormiga 2007). Eye sizes were measured as the maximum dorsal diameter. Leg measurements are shown as total length (femur, patella and tibia, metatarsus, tarsus). All measurements are in millimetres. Specimens examined here are deposited at the School of Life Sciences, Southwest University, Chongqing, China (SWUC).

Abbreviations used in the text: ALE, anterior lateral eye; AME, anterior median eye; B, bursae; CD, copulatory duct; CH, clypeal height; CO, copulatory opening; CT, connecting tube; DTA, dorsal tibial apophysis; E, embolus; FA, femoral apophysis; FD, fertilisation duct; MOA, median ocular area; MS, Median septum; PLE, posterior lateral eye; PME, posterior median eye; PTA, prolateral tibial apophysis; RTA, retrolateral tibial apophysis; SD, sperm duct; TA, tegular apophysis. Spination: d, dorsal; v, ventral; pl, prolateral; pv, prolateral ventral; rv, retrolateral ventral.

## Taxon treatments

### Otacilia baimashan

Shi, Wang & Mu sp. nov.

E969A7BB-2220-5189-B270-5C67017AA26D

F608C7EC-652F-4ED1-9457-FABBCE7C6CBA

#### Materials

- **Type status:** Holotype. **Occurrence:** individualCount: 1; sex: male; lifeStage: adult; occurrenceID: D413F489-A16C-52ED-814D-6D01EDA0CFDE; **Taxon:** scientificName: *Otacilia baimashan* Shi, Wang & Mu, sp. nov.; order: Araneae; family: Phrurolithidae; genus: Otacilia; **Location:** country: China; municipality: Chongqing; locality: Baima Mountain Nature Reserve, Baima Town, Hongmiao; verbatimElevation: 1478; verbatimLatitude: 29°14′58″N; verbatimLongitude: 107°33′47″E; **Event:** year: 2025; month: 9; day: 16; habitat: leaf litter; **Record Level:** institutionID: the Collection of Spiders, Southwest University; institutionCode: SWUC
- **Type status:** Paratype. **Occurrence:** individualCount: 40; sex: 22 male, 18 female; lifeStage: adult; occurrenceID: 1149A4D8-354F-556B-A7C3-9C827E289867; **Taxon:** scientificName: *Otacilia baimashan* Shi, Wang & Mu, sp. nov.; order: Araneae; family: Phrurolithidae; genus: Otacilia; **Location:** country: China; municipality: Chongqing; locality: Baima Mountain Nature Reserve, Baima Town, Hongmiao; verbatimElevation: 1478; verbatimLatitude: 29°14′58″N; verbatimLongitude: 107°33′47″E; **Event:** year: 2025; month: 9; day: 16; habitat: leaf litter; **Record Level:** institutionID: the Collection of Spiders, Southwest University; institutionCode: SWUC
- **Type status:** Paratype. **Occurrence:** individualCount: 8; sex: 6 male, 2 female; lifeStage: adult; occurrenceID: 575C960C-1ED7-5BC7-9676-164EC3331F2F; **Taxon:** scientificName: *Otacilia baimashan* Shi, Wang & Mu, sp. nov.; order: Araneae; family: Phrurolithidae; genus: Otacilia; **Location:** country: China; municipality: Chongqing; locality: Baima Mountain Nature Reserve, Baima Mountain; verbatimElevation: 1478; verbatimLatitude: 29°14′37″N; verbatimLongitude: 107°33′52″E; **Event:** year: 2025; month: 9; day: 16; habitat: leaf litter; **Record Level:** institutionID: the Collection of Spiders, Southwest University; institutionCode: SWUC
- **Type status:** Paratype. **Occurrence:** individualCount: 1; sex: female; lifeStage: adult; occurrenceID: D7315F8A-1580-5016-9C2D-E2E9F1733863; **Taxon:** scientificName: *Otacilia baimashan* Shi, Wang & Mu, sp. nov.; order: Araneae; family: Phrurolithidae; genus: Otacilia; **Location:** country: China; municipality: Chongqing; locality: Baima Mountain Nature Reserve, Waiba Yakou; verbatimElevation: 1478; verbatimLatitude: 29°14′37″N; verbatimLongitude: 107°33′52″E; **Event:** year: 2025; month: 9; day: 16; habitat: leaf litter; **Record Level:** institutionID: the Collection of Spiders, Southwest University; institutionCode: SWUC

#### Description

**Figure 1.**
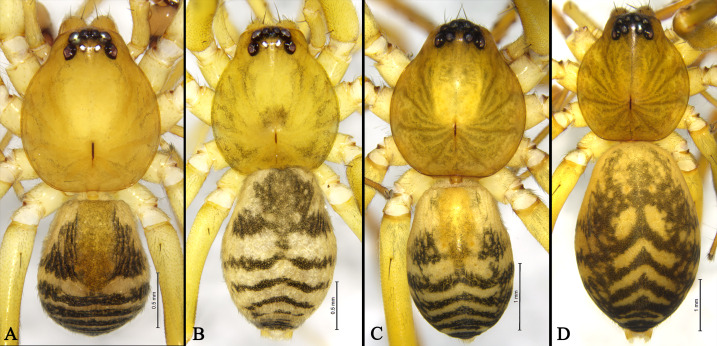
Habitus of *Otacilia baimashan* Shi, Wang & Mu, sp. nov.: **A** Male habitus, dorsal view; **B** Female habitus, dorsal view. Habitus of *Otacilia wulong* Shi, Wang & Mu, sp. nov.: **C** Male habitus, dorsal view; **D** Female habitus, dorsal view.

Male (holotype) total length 2.83 (Fig. 1A and Fig. 4A): carapace 1.50 long, 1.28 wide; opisthosoma 1.29 long, 1.02 wide. Eye sizes and interdistances: AME 0.08, ALE 0.11, PME 0.06, PLE 0.09; AME–AME 0.03, AME–ALE 0.01, PME–PME 0.08, PME–PLE 0.08, ALE–PLE 0.07. MOA 0.21 long, anterior width 0.18, posterior width 0.20. Clypeal height 0.11, CH/AME 1.38. Leg measurements: I 4.88 (1.26, 1.82, 1.17, 0.63); II 4.17 (1.19, 1.42, 0.96, 0.60); III 3.80 (1.01, 1.19, 1.00, 0.60); IV 5.38 (1.38, 1.66, 1.48, 0.86). Leg formula: 4123. Leg spination: femur I d 2 pl 3, femur II d 1 pl 1, femora III–IV d 1; tibia I v 6, tibia II pv 6 rv 5; metatarsus I–II pv 4 rv 3.

Colouration and pattern (Fig. 1A). Carapace yellow, smooth; fovea short, longitudinal; cervical and radial grooves indistinct. Legs yellow, without annulations. Abdomen dorsally yellow, with a small anterior dorsal scutum flanked by two triangular black patches; posterior part with seven transverse, arcuate black bands.

**Figure 2.**
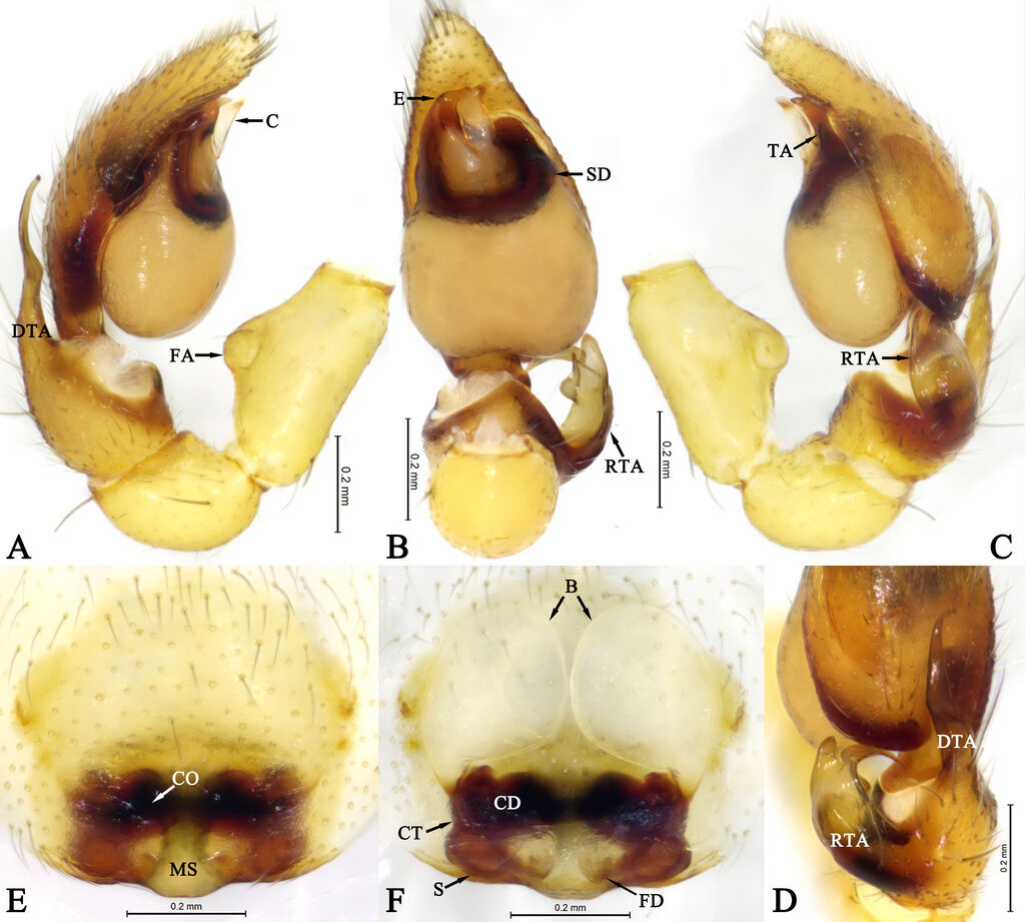
Copulatory organs of *Otacilia baimashan* Shi, Wang & Mu, sp. nov.: **A** Male left male palp, prolateral view; **B** Same, ventral view; **C** Same, retrolateral view; **D** Same, dorsal view; **E** Epigyne, ventral view; **F** Vulva, dorsal view.

Palp (Fig. 2A–D). Femoral apophysis distinct, located at middle part of femur (Fig. 2A and C). RTA base broad, with a ventral tubercle in ventral view (Fig. 2B); distally with a small lamellar branch on prolateral side (Fig. 2C and D), retrolateral branch claw-like and curved towards cymbium (Fig. 2B and D). Dorsal tibial apophysis tapering from base to tip, with tip curving prolaterally in dorsal view (Fig. 2D). Tegular apophysis laminar, arc-shaped (Fig. 2B and C). Sperm duct U–shaped, tapering from retrolateral of tegelum to embolus. Embolus thick at base, curving at 90° at middle part and tapering to tip in ventral view (Fig. 2B). Conductor membranous, translucent (Fig. 2B).

Female (paratype): total length 3.20 (Fig. 1B and Fig. 4B): carapace 1.57 long, 1.34 wide; abdomen 1.72 long, 1.23 wide.Eye sizes and interdistances: AME 0.09, ALE 0.12, PME 0.06, PLE 0.09; AME–AME 0.03, AME–ALE 0.01, PME–PME 0.08, PME–PLE 0.09, ALE–PLE 0.07. MOA 0.22 long, anterior width 0.19, posterior width 0.20. Clypeus height 0.10, CH/AME 1.11. Leg measurements: Ⅰ 5.08 (1.31, 1.94, 1.27, 0.56), Ⅱ 4.50 (1.24, 1.56, 1.07, 0.63), Ⅲ 3.97 (1.09, 1.16, 1.03, 0.69), Ⅳ 5.77 (1.50, 1.76, 1.56, 0.95). Leg formula: 4123. Leg spination: femur I d 2 pl 3, femur II d 1 pl 2, femora III–IV d 1; tibia I v 6 pairs, tibia II pv 6 rv 5; metatarsus I–II pv 4 rv 3.

Colouration and pattern (Fig. 1B). Carapace yellow, smooth, fovea longitudinal, cervical and radial grooves indistinct; with several markings resembling flowing water droplets beside fovea. Other abdomen characters as in male, except without a dorsal scutum.

Epigyne (Fig. 2E and F). Epigynal plate weakly sclerotised. Median septum wide at base. Copulatory openings situated at posterior margin of atrium. Copulatory ducts thick, short; connecting tubes shorter than copulatory ducts. Glandular appendages absent. Bursae balloon–shaped, somewhat translucent. Spermathecae oval, widely separated. Fertilisation ducts located at posterior of spermathecae.

#### Diagnosis

This new species resembles *Otacilia hamata* (Wang, F. Zhang & Z. S. Zhang, 2012) (Wang et al. 2012) in having similar short embolus, membranous conductor and the absence of glandular appendages, but can be recognised by: 1) RTA broad (vs. relatively narrow in *O. hamata*); 2) median septum wide (vs. thin in *O. hamata*); 3) spermathecae oval, separated by about three times their diameter (vs. nearly spherical, separated by their diameter in *O. hamata*).

#### Etymology

The specific name is derived from the type locality; noun in apposition.

#### Distribution

Known only from the type locality, China (Chongqing).

### Otacilia wulong

Shi, Wang & Mu sp. nov.

BF4A3540-D687-59E1-A2B5-7A901DB8D01C

23F2A8BD-C396-4DE7-AAB2-F4F9C7DC76CB

#### Materials

- **Type status:** Holotype. **Occurrence:** individualCount: 1; sex: male; lifeStage: adult; occurrenceID: B93AB4C1-E094-5161-9D1C-2F5C4C5C030C; **Taxon:** scientificName: *Otacilia wulong* Shi, Wang & Mu, sp. nov.; order: Araneae; family: Phrurolithidae; genus: Otacilia; **Location:** country: China; municipality: Chongqing; locality: Baima Mountain Nature Reserve, Baima Town, Hongmiao; verbatimElevation: 1478; verbatimLatitude: 29°14′58″N; verbatimLongitude: 107°33′47″E; **Event:** year: 2025; month: 9; day: 16; habitat: leaf litter; **Record Level:** institutionID: the Collection of Spiders, Southwest University; institutionCode: SWUC
- **Type status:** Paratype. **Occurrence:** individualCount: 20; sex: 13 male, 7 female; lifeStage: adult; occurrenceID: AA0882B9-5DF0-5256-A2C0-2A6C9DD7A413; **Taxon:** scientificName: *Otacilia wulong* Shi, Wang & Mu, sp. nov.; order: Araneae; family: Phrurolithidae; genus: Otacilia; **Location:** country: China; municipality: Chongqing; locality: Baima Mountain Nature Reserve, Baima Town, Hongmiao; verbatimElevation: 1478; verbatimLatitude: 29°14′58″N; verbatimLongitude: 107°33′47″E; **Event:** year: 2025; month: 9; day: 16; habitat: leaf litter; **Record Level:** institutionID: the Collection of Spiders, Southwest University; institutionCode: SWUC
- **Type status:** Paratype. **Occurrence:** individualCount: 6; sex: 1 male, 5 female; lifeStage: adult; occurrenceID: AC7A557A-A214-580D-A65D-F48FF1F76848; **Taxon:** scientificName: *Otacilia wulong* Shi, Wang & Mu, sp. nov.; order: Araneae; family: Phrurolithidae; genus: Otacilia; **Location:** country: China; municipality: Chongqing; locality: Baima Mountain Nature Reserve, Baima Mountain; verbatimElevation: 1484; verbatimLatitude: 29°14′37″N; verbatimLongitude: 107°33′52″E; **Event:** year: 2025; month: 9; day: 16; habitat: leaf litter; **Record Level:** institutionID: the Collection of Spiders, Southwest University; institutionCode: SWUC
- **Type status:** Paratype. **Occurrence:** individualCount: 6; sex: 3 male, 3 female; lifeStage: adult; occurrenceID: 2BF25EE1-B033-53F7-84DE-EA9A13A18A48; **Taxon:** scientificName: *Otacilia wulong* Shi, Wang & Mu, sp. nov.; order: Araneae; family: Phrurolithidae; genus: Otacilia; **Location:** country: China; municipality: Chongqing; locality: Baima Mountain Nature Reserve, Baima Mountain; verbatimElevation: 1474; verbatimLatitude: 29°14′32″N; verbatimLongitude: 107°33′52″E; **Event:** year: 2025; month: 9; day: 16; habitat: leaf litter; **Record Level:** institutionID: the Collection of Spiders, Southwest University; institutionCode: SWUC
- **Type status:** Paratype. **Occurrence:** individualCount: 53; sex: 33 male, 20 female; lifeStage: adult; occurrenceID: CC9716A6-3FF3-5A19-BF3F-9ABEDDB8FDEE; **Taxon:** scientificName: *Otacilia wulong* Shi, Wang & Mu, sp. nov.; order: Araneae; family: Phrurolithidae; genus: Otacilia; **Location:** country: China; municipality: Chongqing; locality: Baima Mountain Nature Reserve, Baima Town, Chengmen Cave; verbatimElevation: 1238; verbatimLatitude: 29°19′24″N; verbatimLongitude: 107°36′58″E; **Event:** year: 2025; month: 9; day: 16; habitat: leaf litter; **Record Level:** institutionID: the Collection of Spiders, Southwest University; institutionCode: SWUC
- **Type status:** Paratype. **Occurrence:** individualCount: 8; sex: 5 male, 3 female; lifeStage: adult; occurrenceID: ED97215F-A3DE-5E6B-8E08-172FA6F3AA52; **Taxon:** scientificName: *Otacilia wulong* Shi, Wang & Mu, sp. nov.; order: Araneae; family: Phrurolithidae; genus: Otacilia; **Location:** country: China; municipality: Chongqing; locality: Baima Mountain Nature Reserve, Baima Town, Shanshuwan; verbatimElevation: 1300; verbatimLatitude: 29°17′20″N; verbatimLongitude: 107°37′12″E; **Event:** year: 2025; month: 9; day: 16; habitat: leaf litter; **Record Level:** institutionID: the Collection of Spiders, Southwest University; institutionCode: SWUC
- **Type status:** Paratype. **Occurrence:** individualCount: 7; sex: 3 male, 4 female; lifeStage: adult; occurrenceID: 56633D34-DD8C-5C8D-9442-D481E6407988; **Taxon:** scientificName: *Otacilia wulong* Shi, Wang & Mu, sp. nov.; order: Araneae; family: Phrurolithidae; genus: Otacilia; **Location:** country: China; municipality: Chongqing; locality: Baima Mountain Nature Reserve, Baima Town, Daquan Village; verbatimElevation: 1363; verbatimLatitude: 29°16′36″N; verbatimLongitude: 107°36′42″E; **Event:** year: 2025; month: 9; day: 16; habitat: leaf litter; **Record Level:** institutionID: the Collection of Spiders, Southwest University; institutionCode: SWUC

#### Description

Male (holotype) total length 4.80 (Fig. 1C and Fig. 4C): carapace 2.36 long, 2.06 wide; abdomen 2.32 long, 1.61 wide. Eye sizes and interdistances: AME 0.16, ALE 0.14, PME 0.12, PLE 0.14; AME–AME 0.05, AME–ALE 0.01, PME–PME 0.17, PME–PLE 0.09, ALE–PLE 0.11. MOA 0.38 long, front width 0.35, back width 0.41. Clypeus height 0.10, CH/AME 1.11. Leg measurements: Ⅰ 9.52 (2.39, 3.65, 2.32, 1.16), Ⅱ 7.82 (2.13, 2.79, 1.78, 1.12), Ⅲ 6.80 (1.88, 2.08, 1.74, 1.10), Ⅳ 10.42 (2.90, 3.16, 2.85, 1.51). Leg formula: 4123. Leg spination: femur I d 2 pl 2, femur II d 1 pl 2, femora III–IV d 1; tibia I pv 8 rv 7, tibia II v 7; metatarsus I v 4, metatarsus II pv 4 rv 3.

Colouration and pattern (Fig. 1C). Carapace light brown, highest near fovea; fovea dark red; cervical grooves indistinct, radial grooves distinct. Legs yellow, without annulations. Abdomen yellow, with a small, triangular dorsal scutum flanked by two triangular black patches posteriorly; posterior part with four chevron‑shaped markings.

**Figure 3.**
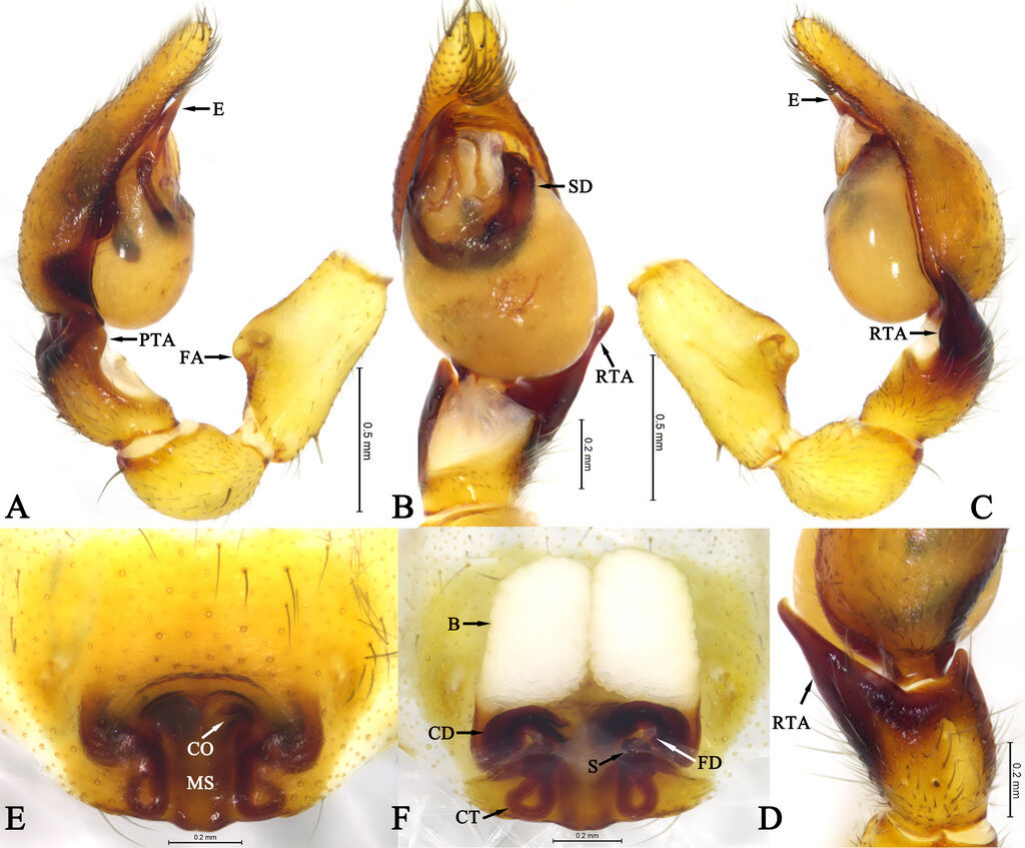
Copulatory organs of *Otacilia wulong* Shi, Wang & Mu, sp. nov.: **A** Male left male palp, prolateral view; **B** Same, ventral view; **C** Same, retrolateral view; **D** Same, dorsal view; **E** Epigyne, ventral view; **F** Vulva, dorsal view.

Palp (Fig. 3A-D). Femoral apophysis distinct, near tip of femur (Fig. 3A and C). RTA base broad, tapering to blunt tip (Fig. 3C and D); prolateral tibial apophysis distinct, rectangular (Fig. 3A). Bulb oval. Sperm duct U–shaped, tapering from retrolateral to embolus. Embolus base broad, upward, tapering from base to tip, with tip extending towards retrolateral margin of cymbium (Fig. 3B). Anterior of tegulum with a transparent membranous area at middle part; conductor absent.

**Figure 4.**
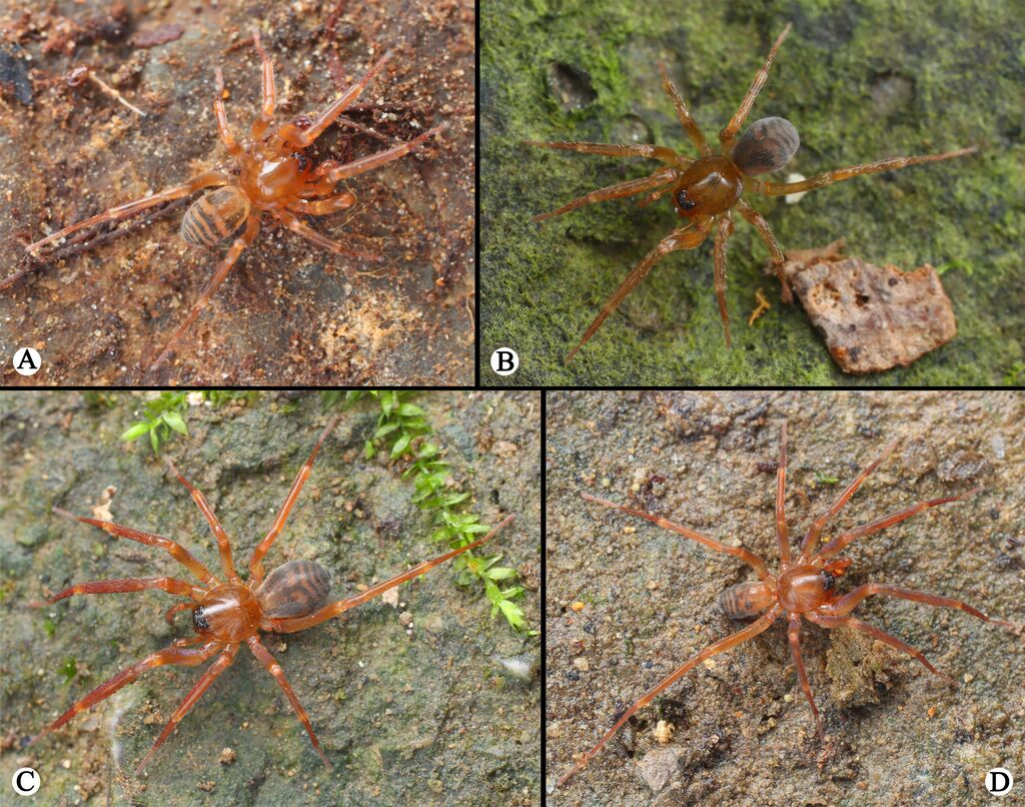
Living photos of *Otacilia baimashan* Shi, Wang & Mu, sp. nov.: **A** Male; **B** Female. Living photos of *Otacilia wulong* Shi, Wang & Mu, sp. nov.: **C** Male; **D** Female. (photographs by Qianle Lu).

Female (paratype): total length 6.24 (Fig. 1D and Fig. 4D): carapace 2.50 long, 2.22 wide; abdomen 3.73 long, 2.47 wide. Eyes. Diameters and interdistances: AME 0.16, ALE 0.17, PME 0.15, PLE 0.17; AME–AME 0.04, AME–ALE 0.02, PME–PME 0.16, PME–PLE 0.10, ALE–PLE 0.13. MOA 0.41 long, front width 0.35, back width 0.44. Clypeus height 0.19, CH/AME 1.12. Leg measurements: Ⅰ 10.14 (2.55, 3.97, 2.42, 1.20), Ⅱ 8.28 (2.13, 3.03, 1.95, 1.17), Ⅲ 7.07 (1.89, 2.22, 1.89, 1.07), Ⅳ 10.81 (2.93, 3.34, 3.03, 1.51). Leg formula: 4123. Leg spination: femur I d 2 pl 4, femur II d 2 pl 2, femora III–IV d 1; tibia I v 8, tibia II pv 7/8 rv 7; metatarsus I v 4 pairs, metatarsus II pv 4 rv 3.

Colouration and pattern (Fig. 1D). Dorsal scutum absent in female. Other characters as in male, except darker colouration.

Epigyne (Fig. 3E and F). Epigynal plate sclerotised, with atrium posteriorly. Copulatory openings small, located at lateral margins of atrium. Median septum rectangular. Copulatory ducts thicker than connecting tubes, transverse, connected to bursae. Connecting tubes slender, coiled into a triangular loop. Glandular appendages absent. Bursae nearly rectangular. Spermathecae spherical, separated by about two spermathecal diameters; fertilisation ducts located at anterior of spermathecae.

#### Diagnosis

This new species resembles *Otacilia simianshan* Zhou, Wang & Zhang, 2013 (Zhou et al. 2013) in having membranous area on anterior of tegulum, but can be recognised by: 1) RTA narrow, tapering from base to tip (vs. RTA wide, broadening from base to tip in *O. simianshan*); 2) embolus slant, extending towards 1 o'clock position (vs. transverse, extending towards 3 o'clock position in *O. simianshan*); 3) epigynal plate with median septum (vs. median septum absent in *O. simianshan*).

#### Etymology

The species name refers to the type locality; noun in apposition.

#### Distribution

Known only from the type locality, China (Chongqing).

## Supplementary Material

XML Treatment for Otacilia baimashan

XML Treatment for Otacilia wulong
